# Oral Care Awareness and Factors Related to the Burden of Nurses at a Community Hospital in Japan

**DOI:** 10.3390/healthcare10061073

**Published:** 2022-06-09

**Authors:** Takashi Koike, Ryuichi Ohta, Yuhei Matsuda, Chiaki Sano, Takahiro Kanno

**Affiliations:** 1 Department of Oral and Maxillofacial Surgery, Unnan City Hospital, 96-1 Daito-cho Iida, Unnan 699-1221, Japan; 2 Department of Oral and Maxillofacial Surgery, Faculty of Medicine, Shimane University, Izumo 693-8501, Japan; matsltoh@tmd.ac.jp (Y.M.); tkanno@med.shimane-u.ac.jp (T.K.); 3 Department of Community Care, Unnan City Hospital, Unnan 699-1221, Japan; ryuichiohta0120@gmail.com; 4 Department of Community Medicine Management, Faculty of Medicine, Shimane University, Izumo 693-8501, Japan; sanochi@med.shimane-u.ac.jp; 5 Department of Lifetime Oral Health Care Science, Graduate School of Medical and Dental Sciences, Tokyo Medical and Dental University, Tokyo 113-8510, Japan

**Keywords:** mouth, care, nurses, community hospital, burden

## Abstract

Objective: This cross-sectional study investigated the oral care knowledge, awareness, and challenges of 159 nurses and identified the factors related to the burden of oral care in Unnan City Hospital, Japan. Materials and Methods: This study included outpatient, ward, and operating room nurses who answered a questionnaire comprising 19 questions regarding awareness, actual implementation status of oral care provided, burden and learning experiences of oral care, and participants’ characteristics. A univariate regression model was used to assess whether catheterization was associated with the independent variables. Results: The number and rate of valid questionnaires were 134 and 87.6%, respectively. The mean years of clinical experience were 18.71 ± 12.02 years; 95.5% of the nurses were women. There were significant differences in the Oral Health-related Caregiver Burden Index among “interest in oral care” (*p* = 0.006), “priority of oral care” (*p* = 0.005), and “burden of oral care”. Conclusions: This study shows that nurses who are highly interested in oral care, prioritize oral care, and do not perceive oral care as a burden. Educational interventions, such as training sessions and direct guidance to solve challenges, are needed and can lead to improvements in the quality of life and advancement of health.

## 1. Introduction

The importance and need for oral care in whole-body management have been recognized. As aspiration pneumonia in elderly individuals is becoming a critical challenge, several studies regarding the effects of oral care on the prevention of aspiration pneumonia have been published [1,2]. In addition, the implementation of advanced oral care decreases the frequency of fever and reduces the risk of pneumonia, improving activities of daily living (ADLs) [3,4]. For instance, maintenance of oral health conditions in patients with malignant tumors at the time of surgery, radiation therapy, and chemotherapy are helping to improve prognosis and prevent complications [5,6].

Nationwide efforts have been made to manage perioperative oral functions. In patients with cardiac diseases, treatment of ventilator-associated pneumonia in the intensive care unit and maintenance of oral health conditions in unawakened patients are required [7,8]. In patients with cerebrovascular diseases, residual disabilities such as paralysis and dysphagia should be managed. Additionally, it is necessary to deal with refusal of oral care in patients with dementia [9,10,11]. Improvement of the oral environment, including oral functions under various circumstances, is needed [1,2,3,4,5,6,7,8,9,10,11].

In Japan, hospitals have a high proportion of elderly individuals aged 65 years or above and there are various challenges regarding oral health. Especially, many hospitalized patients are the oldest elderly people in community hospitals [12]. Older individuals tend to experience a decline in ADLs and swallowing function, a decrease in saliva production, and low resistance to infection. These health issues could cause aspiration of food and saliva and reduced self-cleaning function, aggravating the oral environment. Moreover, the oldest elderly people are unable to perform oral health care by themselves because of their diseases and disabilities. Thus, healthcare workers perform oral care management for them. However, most nurses are unable to allow sufficient time for such oral care management currently as they are routinely busy and prioritize whole-body management over oral care. Furthermore, many nurses are unable to provide adequate oral care because they lack a complete understanding of the need and methods of oral care [13,14,15]. Before our department was established, accessing specialist dental care was difficult. To date, nurses and speech-language pathologists have felt anxious about providing oral care because they do not receive sufficient support and management from their hospital. In addition, there was no unified method for oral care, and these professionals practice oral care based on information provided in nursing magazines and seminars.

The importance of oral care in nursing services has been recently recognized. In 2006, the Japanese Nursing Association started the nurse certification system for certified nurses in dysphagia nursing, and oral care is included in its training program. Therefore, oral care is positioned as essential care for hospitalized patients in nursing services [15].

Additionally, ward nurses consider that intervention by dental/oral specialists is necessary for oral care for hospitalized patients [13]. Prior literature shows that the intervention of professional oral care leads to the reduction of oral and periodontal bacteria. Moreover, it leads to the modification of the consciousness of hospital staff regarding oral care. These results indicated that a professional oral care system under close cooperation between professionals might lead to a favorable outcome [16]. Another study shows that oral management by dental hygienists improves patient outcomes, including ADLs, home discharge, and in-hospital mortality in post-acute rehabilitation [17]. From the results of these surveys, it is suggested that professional care by dental hygienists is required to improve oral hygiene management and feeding functions of bedridden elderly individuals. However, because of a lack of time and manpower, it is difficult for dentists to provide oral care to all hospitalized patients. Thus, both routine oral care by ward nurses and advanced care by dental/oral specialists are necessary for hospitalized patients. We focused on the burden of oral care and oral health management being viewed as key to its improvement. Moreover, we hypothesized that a reduction in nurses’ burden of oral care can increase the frequency of oral care and that frequent oral care can contribute to improvement in patients’ systemic health outcomes.

Therefore, for both nurses and dental/oral professionals to provide high-quality oral care, it is important to understand the current state of oral care among nurses, their knowledge and awareness of oral care, and challenges in medical clinical settings. Previous studies reported that the general caregiver burden correlates with the oral health-related caregiver burden. In the present study, we performed a questionnaire survey of all the nurses in our hospital to identify problems and factors related to the burden of oral care.

## 2. Materials and Methods

### 2.1. Participants

We surveyed 159 nurses at Unnan City Hospital, including outpatient, ward, and operating room nurses. This hospital is located in Unnan City, in the mountainous area of Shimane Prefecture, Japan. Unnan City has a population of 37,000 and its population aging rate is 39.95%. This general hospital has 281 beds and 15 departments and is also a regional referral hospital in the city.

### 2.2. Study Design

This was a cross-sectional study.

### 2.3. Survey Methods

Our survey was performed using a questionnaire method. We distributed a questionnaire to participants, and the questionnaire was filled out anonymously. We prepared a unique questionnaire based on previous studies to ensure the credibility and reasonability of the questions [13,14,15,18,19,20,21,22,23,24]. With reference to those studies, we classified the important domains that make up the research questions, and created a comprehensive questionnaire by listing as many question proposals as possible regarding each domain. Following this, the less important ones were verified and removed to complete it. In addition, as a preliminary test, the nursing director attempted the questionnaire and verified whether the respondents could read, interpret, and answer it according to the intended meaning. The questionnaire comprised 19 questions, including those regarding awareness, the actual implementation status of oral care provided by nurses, burden and learning experience regarding oral care, and participants’ characteristics.

We explained our research purpose and contents to the director of nursing and obtained consent for the survey. We attached a request and consent form to the questionnaire. The request form included the research purpose and method and stated that those who did not participate in the survey suffered no disadvantages and that the data are used for only research purposes to respect participants’ privacy. The questionnaire was distributed to all nurses by the nursing director. After consulting with the nursing department, we collected the responses after three weeks. To respect participants’ privacy, we separated the consent form from the questionnaire after collection. Participants were informed that they could withdraw their participation at any time, for any reason, and without repercussions. Moreover, the data were accessible only to the authors. The survey results were anonymized. This study was approved by the Unnan City Hospital Ethical Committee (approval number: 20200021) to guarantee the rights of the research subjects based on ethical principles.

### 2.4. Data Collection Procedures

We distributed 159 questionnaires and collected 153 (96.2% collected). Incomplete questionnaires such as those containing unanswered question items were invalid. The number of valid questionnaires was 134, and the proportion of valid questionnaires collected was 87.6%. We tallied the number of the answers from 134 nurses regarding the awareness, burden, learning experience about oral care, and participants’ characteristics and analyzed their proportions (Table 1). Additionally, we surveyed nurses regarding the actual implementation status of oral care, such as the average time spent on oral care for each patient, the number of times oral care was performed in a day, and the ideal number of times. If there was a range in the frequency of oral care, we used the maximum value for analysis. A prior study developed the Oral Health-related Caregiver Burden Index (OHBI) to evaluate the burden of oral care and showed that the general caregiver burden correlates with the oral health-related caregiver burden [25]. We applied the OHBI to our survey of nurses. The OHBI has one question item about oral care burden and nine question items regarding the following four domains of burden: technique-related, service-related, existential, and risk-related burdens. Participants were divided into two groups using the median of each item’s score, and the groups were compared in consideration of their backgrounds, such as years of clinical experience.

### 2.5. Statistical Analysis

The Student’s *t*-test was performed on parametric data, and the Mann–Whitney U test was performed on non-parametric data. Numerical variables were dichotomized as follows: learning interest (strong or a little = 1; no relative interest, no interest, or no idea = 0); need for oral care (strong or a little = 1, no relative interest, no interest, no idea = 0); and priority of oral care (very high or relatively high = 1, not high, not relatively high, or no idea = 0). Regarding the OHBI, participants were divided into two groups according to their total OHBI being above or below the median (25). To clarify the statistical difference in the rate of fatigue of oral care between the two groups, a minimum of 50 participants were required in each group, based on α (alpha) = 0.05, β (beta) = 0.20 and a power of 80%. A univariate regression model was used to assess whether performing catheterization was associated with the independent variables. Cases with missing data were eliminated from the analysis. Statistical significance was defined as a *p* < 0.05. All statistical analysis was performed using EZR (Saitama Medical Center, Jichi Medical University, Saitama, Japan), which is a graphical user interface for R (The R Foundation, Vienna, Austria).

## 3. Results

### 3.1. Participant Characteristics

The mean years of clinical experience were 18.71 ± 12.02 years, and 95.5% of nurses were women. Approximately 81.3% of them were ward nurses and the others were outpatient or operating room nurses (Table 2).

### 3.2. Awareness of Oral Care

Regarding the interest in oral care, 73.9% of nurses answered “strong” or “a little”. Moreover, regarding the necessity of oral care, 98.5% of nurses answered “strong” or “a little”, indicating that almost all nurses were aware of the necessity of oral care. For the effect of oral care, most nurses understood that oral care is helpful for the prevention of not only local infections but also systemic infections. Only 30.6% of nurses answered “very high” or “relatively high” regarding the priority of oral care compared to care for other parts of the body (Table 2).

### 3.3. Actual Implementation Status of Oral Care

The average time spent on oral care in each patient was 4.76 ± 2.99 min, 2.35 ± 0.99 times a day. In contrast, nurses answered that they needed to provide oral care an average of 3.25 ± 0.91 times a day, and only 8.65% answered that they were “very much satisfied” or “satisfied” with their oral care.

### 3.4. Burden of Oral Care

In the analysis, background factors including age, gender, interest in oral care, need for oral care, the priority of oral care, participants’ department, and learning experience were compared between groups (Table 3). The results showed a significant difference (*p* < 0.05) in “interest in oral care”, and “priority of oral care”. This suggests that nurses who were highly interested in oral care and experiential learning and those who prioritized oral care had a tendency not to feel oral care was a burden (Table 4).

### 3.5. Learning Experience about Oral Care

In the question regarding learning about oral care, 73.9% of nurses answered that they had prior learning experience. The options for the learning methods were “nursing school class”, “training session for nurses”, and “seminar” (Table 2). 

## 4. Discussion

To the best of our knowledge, our study is the first to identify the factors involved in the burden of oral care by using statistical analyses in a regional community hospital in Japan. The OHBI was developed in a previous study, and the general caregiver burden correlates with that of the oral health-related caregiver burden. This suggests that the oral health-related caregiver burden becomes an obstacle to oral care. It also reported that it is necessary to reduce the burden of promoting oral care in facilities for elderly people. These results suggest that a reduction in the burden of oral care increases the frequency of oral care [25]. Therefore, we therefore hypothesized that a reduction in nurses’ burden of oral care could increase the frequency of oral care and that can contribute to improvement in patients’ systemic health outcomes. Educational guidance to reduce the burden may increase the frequency and improve quality. However, commitment to quality may generate negative feedback to increase the burden, resulting in a decrease in frequency. Thus, we focused on increasing frequency by reducing the burden. A study conducted at another facility reported that nurses are unable to spend sufficient time on oral care since they have to prioritize whole-body management over oral care in daily services under present circumstances. This study also showed that 81.0% of nurses answered “sometimes”, “quite frequently”, or “nearly always”, and “I have no time to provide oral care service” in response to the question about oral care burden. Only 30.7% of nurses answered “high” or “very high” to the question about the priority of oral care over that of other parts of the body, although they had great interest and necessity. The results from our statistical analyses using the OHBI showed that there is no significant association between “participants’ high level of interest in oral care”, “participants’ high priority to oral care”, and burden. Nurses who were highly interested in oral care and prioritized oral care might not feel oral care to be a burden. This suggests that there is an association between “interest”, “priority”, and “burden”. These results suggest that educational interventions such as training sessions and direct instructions may be necessary for nurses to have a high interest in oral care and to prioritize it. Additionally, we need to prove that there is an association between “interest”, “priority”, and “burden”, by performing an intervention study. Even in hospitals without dental/oral professionals, the challenge of nursing management practices for oral health care in the future is to improve the education system that considers the burden on nurses and other care workers and improve cooperation with dental/oral professionals. In addition to these educational interventions, we should consider the introduction of educational programs and the transfer of duties to nursing assistants.

Management of oral hygiene and health has been regarded as being important for whole-body management. Oral care increases patients’ comfort by preventing bad breath. Moreover, it prevents caries and periodontal diseases. In addition to that, oral care is important for the prevention of systemic infections such as aspiration pneumonia [1,2,3,4]. Therefore, the medical significance of oral care has been recognized [1,2,3,4,5,6]. Survey results in the present study showed that 73.9% of nurses had “strong” or “a little” interest in oral care for hospitalized patients. The rate was as high as that in other facilities that performed a similar survey [14,21,23]. Currently, oral care draws a lot of attention, and our results suggest that many nurses have an interest in oral care. In response to the question about the need for oral care, “Do you think that oral care is necessary for hospitalized patients?” approximately 98.5% answered “strong” or “a little”. This indicates that many nurses felt that oral care is necessary, suggesting that participants have high awareness.

Oral health care is a basic nursing technique and nurses perform oral care, particularly for patients who are unable to perform it by themselves. However, nurses are busy and are unable to provide sufficient oral care in many hospitals and medical facilities in the context of rising awareness of oral care [13,14,15]. Answers to the question about the actual implementation status of oral care showed that nurses spent an average of 4.76 ± 2.99 min per day on oral care per patient and provide oral care 2.35 ± 0.99 times a day. Approximately 0.93% of nurses did not perform oral care, suggesting that some nurses did not perform it daily. However, the nurses answered that they needed to provide oral care an average of 3.25 ± 0.91 times a day and only 8.65% answered that they were “very much satisfied” or “satisfied” with their oral care. This rate is quite low, and this result suggests that nurses were so busy that they could not spend much time on oral care in daily services as mentioned above, although they had a high awareness of oral care. 

This study shows that because of time constraints, burden, and lack of knowledge, many nurses have difficulty providing oral care for patients who refuse oral care although nurses understand its importance. However, patients need to keep receiving oral care during hospitalization and after discharge, as oral care is one of their ADLs. Therefore, not only healthcare workers but also patients themselves and their family members need to understand the necessity of oral care, which can lead to improvement in quality of life and advancement of systemic health in patients.

To the question regarding learning about oral care, 73.7% of the nurses answered that they had prior learning experience. There was a tendency among less experienced nurses to answer that they learned about oral care in “nursing school class” and among more experienced to answer that they learned about oral care in “training sessions for nurses” or “seminars”. This suggests that the nursing education program has been modified. A prior study searched a database of The Japan Medical Abstract Society for the articles about “oral care” and reported that there were few articles about oral care in the nursing area before 1990. However, there has been a gradual increase in the number of articles after 1990 and a particularly remarkable increase after 2010. This suggests that the importance of oral care in nursing has been recognized [15] and that nurses are interested in practical knowledge and methods of providing oral health care in medical clinical settings. Additionally, 73.3% of nurses learned about oral care. However, there is a limit to learning for patients with deteriorated oral environments and patients with poor systemic conditions only through nursing school programs and other learning opportunities such as training sessions for nurses and seminars [26]. An appropriate oral assessment is necessary for oral care. Uniform ways of brushing, cleaning of oral and periodontal mucous, moisturizing, cleaning of dentures and oral rinsing are insufficient for such patients. It is necessary to perform procedures suitable for individual patients considering the conditions of teeth, and oral and periodontal mucosa, salivation, dietary intake, and systemic conditions. Therefore, nurses should acquire basic knowledge, methods of oral care, and the capacity to determine if a patient has difficulty in oral care to appropriately refer the patient to a dental/oral department [27,28]. 

To be able to identify oral care difficulties, it is necessary to educate nursing students to develop an interest in oral care by conducting clinical training and lectures on oral care at an early stage. In the future, we plan to develop an educational program and conduct regular study sessions on oral care. Considering oral care in cooperation with experts contributes to the implementation of effective oral care for patients. Therefore, creating an environment that makes cooperation easier and establishing seamless relationships between nurses and dental/oral specialists will be needed.

## 5. Limitations

The limitations of this study are as follows. First, it was not possible to verify whether the prepared questionnaire can properly measure the target of this study without excess or deficiency. We should verify this point in future research. Second, there are only a few male nurses in our hospital. In Japan, the number of male nurses is still smaller than that of female nurses, however, future research should focus on male nurses as well. Third, we did not distinguish between managers, such as the director of the nursing department, and general nursing staff, hence, the difference could not be measured. However, since all managers at regional hospitals work as nursing staff, we believe that there will be no major differences or impact on oral care. Fourth, this study was only conducted in a hospital located in an area with an aging population. The uniqueness of the area and hospital characteristics could have influenced the results. Caution should be exercised when the results are generalized. In this study, we conducted a questionnaire survey of the nurses working in a regional community general hospital in Japan. 

## 6. Conclusions

There are a few reports of questionnaire surveys of nurses on oral care for hospitalized patients. To the best of our knowledge, this study is the first to discuss factors involved in the burden of oral care using statistical analyses. In the future, we plan to develop an effective intervention to solve problems after careful consideration of this study’s results. We concluded that a medical health care team must be established to provide effective oral care with close cooperation between dental/oral professionals and nurses. Moreover, it is important to reduce nurses’ burden and create an environment that facilitates the effortless provision of oral care. 

## Figures and Tables

**Table 1:** Questionnaire items.

Question	Questionnaire Classification	Answer
Are you interested in oral care for hospitalized patients?	Collected questionnaire	Strong, a little, no relative interest, no interest, no idea
Do you think that oral care is necessary for hospitalized patients?	Collected questionnaire	Strong, a little, no relative interest, no interest, no idea
Do you prioritize oral care over the care of other body parts?	Collected questionnaire	Very high, relatively high, not high, not relatively high, no idea
How long at a time do you spend on oral care?	Open-ended questionnaire	( ) minutes per day
How many times a day do you provide oral care?	Open-ended questionnaire	( ) times a day
How many times a day do you think you provide oral care idealistically?	Open-ended questionnaire	( ) times a day
Are you satisfied with your oral care?	Collected questionnaire	Very much satisfied, satisfied, not relatively satisfied, not satisfied, no idea
I have no time to provide oral care services.	Collected questionnaire	Never, rarely, sometimes, quite frequent, nearly always
My body aches when brushing.	Collected questionnaire	Never, rarely, sometimes, quite frequent, nearly always
I want to delegate the oral care to someone else.	Collected questionnaire	Never, rarely, sometimes, quite frequent, nearly always
I experience hardship because caregiving does not give me a sense of satisfaction.	Collected questionnaire	Never, rarely, sometimes, quite frequent, nearly always
I feel endangered when brushing teeth for senior citizens.	Collected questionnaire	Never, rarely, sometimes, quite frequent, nearly always
I do not know what to do about oral care on assistant.	Collected questionnaire	Never, rarely, sometimes, quite frequent, nearly always
I feel uncomfortable because of the unpleasant oral appearance and odor when brushing.	Collected questionnaire	Never, rarely, sometimes, quite frequent, nearly always
I have a hard time because patients resent receiving oral care.	Collected questionnaire	Never, rarely, sometimes, quite frequent, nearly always
Overall, how much burden do you think providing oral health care is to you?	Collected questionnaire	Never, rarely, sometimes, quite frequent, nearly always
Have you ever learned about oral care?	Collected questionnaire	Yes, no
Please tell us how many years of clinical experience you have.	Open-ended questionnaire	( ) years
Please tell us which department you belong to.	Collected questionnaire	Outpatient, ward, operating room

**Table 2:** Demographic data (*n* = 134).

Item	Category	Overall (%)
Gender	Male	8 (4.5)
	Female	126 (95.5)
Age	20–29 years	28 (21.2)
	30–39 years	22 (16.7)
	40–49 years	39 (29.5)
	50–59 years	29 (22.0)
	60–69 years	14 (10.6)
Interest	Strong	9 (6.7)
	A little	90 (67.2)
	No relative interest	25 (18.7)
	No interest	10 (7.5)
	No idea	0 (0)
Necessary	Strong	68 (50.7)
	A little	64 (47.8)
	No relative interest	2 (1.5)
	No interest	0 (0)
	No idea	0 (0)
Priority	Very high	0 (0)
	Relatively high	41 (30.6)
	Not high	58 (43.3)
	Not relatively high	31 (23.1)
	No idea	4 (3.0)
Learning experience	Yes	99 (73.9)
	No	35 (26.1)
Department	Outpatient	20 (14.9)
	Ward	109 (81.3)
	Operating room	5 (3.7)

**Table 3:** Oral Health-related Caregiver Burden Index scores.

Question	Average Value (Standard Deviation)
I have no time to provide oral care services.	3.03 (0.79)
My body aches when brushing.	2.59 (0.91)
I want to delegate the oral care to someone else.	2.96 (0.96)
I experience hardship because caregiving does not give me a sense of satisfaction.	2.43 (0.83)
I feel anxious when brushing senior citizens’ teeth.	2.54 (0.72)
I do not know what to do about oral care on assistant.	2.60 (0.68)
I feel uncomfortable because of the unpleasant oral appearance and odor when brushing.	3.09 (0.87)
I have a hard time because patients resent receiving oral care.	3.26 (0.71)
Overall, how much burden do you think providing oral health care is to you?	2.37 (0.88)
Total	24.87 (4.53)

Answer (score): never (1), rarely (2), sometimes (3), quite frequent (4), nearly always (5).

**Table 4:** Factors related to the burden of oral care in univariate analysis.

Variables	Category	OHBI < 25	OHBI ≥ 25	*p*-Value
		(*n* = 66)	(*n* = 68)	
Gender (%)	Female	61 (95.3)	65 (95.6)	1
Age (%)	20–29 years	12 (18.8)	16 (23.5)	0.278
	30–39 years	14 (21.9)	8 (11.8)	
	40–49 years	18 (28.1)	21 (30.9)	
	50–59 years	16 (25.0)	13 (19.1)	
	60–69 years	4 (6.2)	10 (14.7)	
Interesting (%)	1	56 (84.8)	43 (63.2)	0.006 *
Necessary (%)	1	66 (100.0)	66 (97.1)	0.496
Priority (%)	1	28 (42.4)	13 (19.1)	0.005 *
Learning experience (%)	1	54 (81.8)	45 (66.2)	0.05
Clinical experience (%)	Yes	18.56 (12.07)	18.85 (12.15)	0.891
Department (%)	Outpatient	8 (12.1)	12 (17.6)	0.573
	Ward	54 (81.8)	55 (80.9)	
	Operating room	4 (6.1)	1 (1.5)	

* *p* < 0.05. OHBI, oral health-related caregiver burden index.

## Data Availability

The data that support the findings of this study are available from the corresponding author.
